# The New Medical Society

**Published:** 1878-07

**Authors:** 


THE NEW MEDICAL SOCIETY met at the Washingtonian Home, Monday evening, May 13th, the President, Dr. Bridge, in the chair. Thirty-three ladies and gentlemen were in attendance.

After the admission of ten applicants for membership, Dr. Lyman read a paper on "Fermentation." After briefly reviewing the history of fermentation, the theories that have been advanced to explain its phenomena, he stated the two principle views now urged. One, advocated prominently by Pasteur, maintains that the presence of a vegetable fungus is necessary to the process; that alcohol and carbonic dioxide are excretions of this fungus ofter feeding upon sugar. Yet as these substances have been produced from sugar without the presence of such fungus, Pasteur saves his theory only by denying the right to the name fermentation, of any process not induced by the yeast plant or similar organism.

The other view—the physico-chemical theory, insists that the common fact in all fermentations, is the presence of organic matter in a state of rapid change from complex to simplex forms.

A ferment is defined as "the product of an organized body; its molecules are in a condition of unstable equilibrium and are in a state of violent agitation, by virtue of which they are enabled to profoundly affect the modes of motion which obtain in th molecules of certain organized bodies or products of organizations."

Such rapidly disintegrating substances are found not only in the matter thrown off from the yeast cell (perhaps as an excrement), but also in diastase, ptyalin, pepsin, pancreatine and liver-ferment. These are the products of normal cell-action; a deviation from such normal action furnishes the ferment of hydrophobia, glanders, vaccinia, small-pox, measles, etc.

This idea simplifies the mysteries of infection, for by it we can understand not only the transfer of septic influence, but also the origin of such influence within the individual—a true auto-infection.

Moreover by supposing variations in the stability of molecular equilibrium of ferments, we may explain the variable intensity of the infection of typhoid fever, for example; and in the same supposition see the difference between sporadic and epidemic cholera.

So, too, the fatal persistence with which an unfortunate obstetrician occasionally infects his puerperal patients may be due not to any transfer of septic material from one woman to others, either by hand, clothing or atmosphere, but simply, to an infective process—"fermentation"—in the doctor's own person, which though it be impotent to affect his general health, is sufficient to profoundly impress the puerperal woman whose tissues are predisposed to such molecular disturbance.

Dr. Lyman elaborated at length the chemical theory of fermentation, with special reference to the zymotic diseases, making a very complete and suggestive paper.

In the discussion which followed, Dr. Allen mentioned two very interesting cases in which phthisis had been apparently communicated by infection.

